# Tumor mutational burden predictability in head and neck squamous cell carcinoma patients treated with immunotherapy: systematic review and meta-analysis

**DOI:** 10.1186/s12967-024-04937-x

**Published:** 2024-02-04

**Authors:** Juan P. Rodrigo, Mario Sánchez-Canteli, María Otero-Rosales, Pablo Martínez-Camblor, Francisco Hermida-Prado, Juana M. García-Pedrero

**Affiliations:** 1 Department of Otolaryngology, Hospital Universitario Central de Asturias and Instituto de Investigación Sanitaria del Principado de Asturias (ISPA), Instituto Universitario de Oncología del Principado de Asturias, University of Oviedo, 33011 Oviedo, Spain; 2 Ciber de Cáncer, CIBERONC, 28029 Madrid, Spain; 3 Department of Otolaryngology, Hospital Universitario de Cabueñes and Instituto de Investigación Sanitaria del Principado de Asturias (ISPA), 33011 Oviedo, Spain; 4 Department of Anesthesiology, Dartmouth Hitchcock Medical Center, Geisel School of Medicine at Dartmouth, Lebanon, NH USA; 5 Faculty of Health Sciences, Universidad Autonoma de Chile, 7500912 Providencia, Chile

**Keywords:** Head and neck squamous cell carcinoma, Meta-analysis, Tumor mutational burden, Immune checkpoint inhibitors

## Abstract

**Background:**

Tumor mutational burden (TMB) has been demonstrated to predict the response to immune checkpoint inhibitors (ICIs) in various cancers. However, the role of TMB in head and neck squamous cell carcinoma (HNSCC) has not yet been specifically addressed. Since HNSCC patients exhibit a rather limited response to ICIs, there is an unmet need to develop predictive biomarkers to improve patient selection criteria and the clinical benefit of ICI treatment.

**Methods:**

We conducted a systematic review and meta-analysis according to Preferred Reporting Items for Systematic Reviews and Meta-analyses (PRISMA) reporting guidelines. HNSCC cohort studies were selected when TMB prior to ICI treatment was evaluated, TMB cutoff value was available, and the prognostic value of TMB was evaluated by time-to-event survival analysis. A total of 11 out of 1960 articles were analyzed, including 1200 HNSCC patients.

**Results:**

The results showed that those patients harboring high TMB exhibited a significantly superior overall response rate (OR = 2.62; 95% CI 1.74–3.94; *p* < 0.0001) and a survival advantage (HR = 0.53; 95% CI 0.39–0.71; *p* < 0.0001) after ICI treatment.

**Conclusion:**

This is the first meta-analysis to demonstrate a higher response and clinical benefit from ICI therapy in HNSCC patients with high TMB.

**Graphical Abstract:**

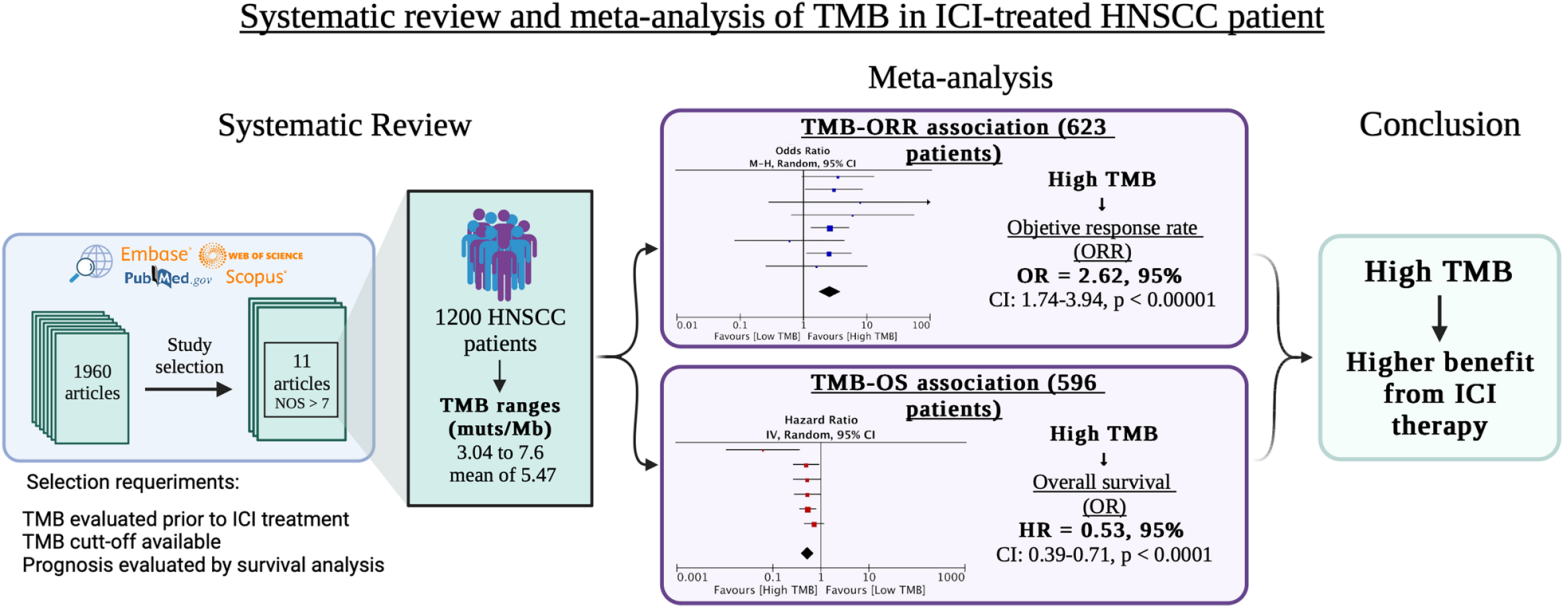

**Supplementary Information:**

The online version contains supplementary material available at 10.1186/s12967-024-04937-x.

## Background

Head and neck squamous cell carcinoma (HNSCC) is the predominant cancer in the head and neck area, and the sixth most frequently malignancy worldwide [1]. In recent decades, the survival rates for HNSCC patients have only modestly improved due to combined treatment modalities, which are mainly surgery, radiotherapy, and chemotherapy. Therefore, there is an imperative need to enhance the prognosis prediction ability for HNSCC patients, to tailor effective individualized therapeutic strategies and to acquire insight into the underlying mechanisms that contribute to treatment response/resistance and therapeutic failure [2].

The intricate interplay between the tumor and the surrounding stroma, the so-called jointly tumor microenvironment (TME), plays a central role in tumor progression and treatment response. The TME encompasses diverse cellular components, such as stromal fibroblasts, endothelial cells, blood vessels, lymph vessels, and immune cells, among others [3]. Immune evasion has been recognized as an emerging hallmark of cancer, and in recent years numerous mechanisms have been identified as promising targets for anti-cancer immunotherapy [4].

Immune checkpoint inhibitors (ICIs) have emerged as a novel promising class of anticancer agents and a central pillar in the treatment of advanced cancers. There is substantial evidence of long-lasting response to ICIs and survival advantage in platinum-pretreated recurrent and metastatic (R/M) HNSCC [5–8]. However, the overall response rates (ORRs) to these agents in platinum-refractory recurrent and metastatic (R/M) HNSCC are as of yet rather modest, ranging from 13 to 18% [5, 9, 10]. Given the significant cost and potential immune-related toxicities associated to ICI therapy, it poses a crucial challenge to identify and validate reliable predictive biomarkers of ICI efficacy to guide patient selection and clinical decision-making. Among the biomarkers for consideration are PD-L1 protein expression, intratumoral immune cell infiltration, immune-gene expression profiling, and tumor mutational burden (TMB). However, none of these biomarkers have been validated in the context of HNSCC to date [10, 11].

The TMB refers to the number of non-synonymous mutations per megabase detected in a tumor exome by next-generation sequencing (NGS). An elevated TMB corresponds to increased neoantigen production by tumor cells, which enhances potential recognition by the immune system. Consequently, ICI-induced antitumor immune responses may facilitate the elimination of tumor cells [12, 13]. A comprehensive analysis integrating 45 clinical studies and data from 103,078 cancer patients revealed that high TMB may serve as an unfavorable prognostic indicator for patients undergoing non-immunotherapy treatment. By contrast, in the subset of patients who received immunotherapy, high TMB was globally found to associate significantly with improved survival and treatment efficacy irrespective of the cancer type [14].

The predictive and prognostic value of TMB in cancer immunotherapy has been validated in certain specific cancer types, such as non-small cell lung cancer, melanoma, and colorectal cancer [13, 15–17]. However, the impact of TMB in HNSCC patients has not been specifically and properly addressed. Even though it has been reported that HNSCC patients with elevated TMB who received immunotherapy showed higher response rates and better prognoses, the studies published to date present several methodological limitations. Overall, the small sample size and lack of consensus on the optimal TMB cutoff value complicate a possible implementation in routine clinical practice [18–28]. This prompted us to undertake the first meta-analysis aimed at evaluating the prognostic and predictive significance of TMB in R/M HNSCC patients treated with ICIs.

## Materials and methods

### Search strategy

We conducted a systematic review through the examination of the current existing literature according to the Preferred Reporting Items for Systematic Review and Meta-Analyses (PRISMA) guidelines [29]. The PRISMA Checklist is included as Additional file 1: Table S1. The overall goal of this search strategy was to identify and include all relevant articles specifically focused on assessing the impact of TMB in HNSCC patient cohorts. This systematic review is registered in Open Science Framework (identifier: https://osf.io/7gmdc).

**Fig. 1:**
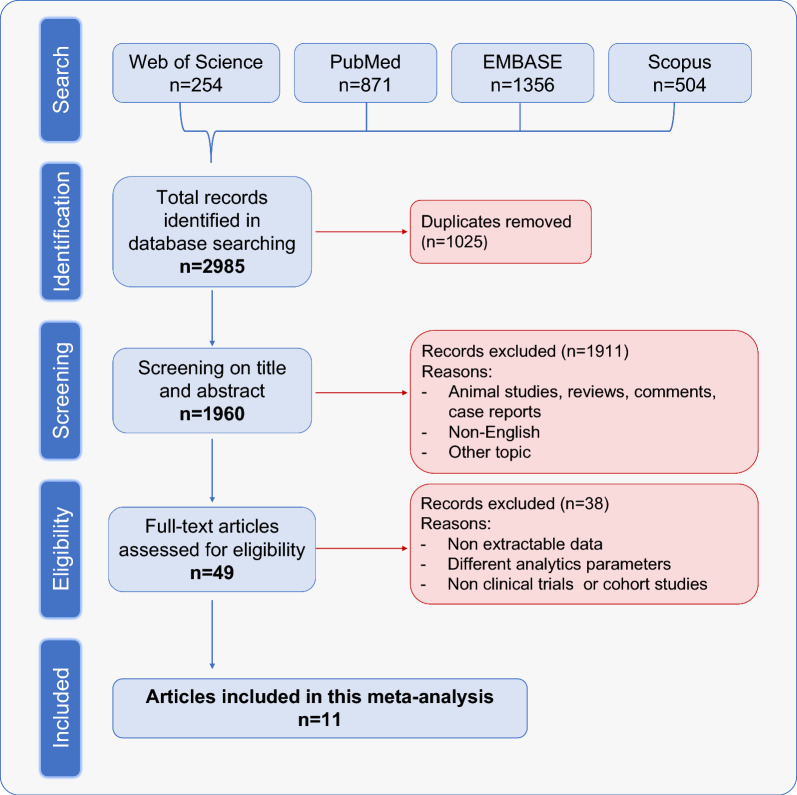
Flow chart depicting the study selection process for the systematic review

An updated PubMed, Embase, Web of Science and Scopus internet search was performed on December 26, 2023, encompassing English language publications from 2011 to 2023, the time period since the first ICI agent was approved by the FDA. The search criteria included the following terms in the title or abstract: "Tumor Mutational Burden" combined with "Head and Neck Cancer" OR "Oral cancer" OR "Oropharyngeal cancer" OR "Laryngeal cancer" OR "Hypopharyngeal cancer" (Additional file 1: Table S2). Two independent researchers (JPR and MS-C) reviewed the search results to identify potentially eligible studies. In those studies where the abstract mentioned follow-up data and outcomes related to TMB in HNSCC, the full-text article was retrieved and reviewed. Additionally, all review articles were thoroughly examined. The references of the retrieved full-text articles were cross-checked to ensure appropriate inclusion in this review (Fig. 1). Any disagreements regarding the eligibility of an article were resolved through consensus.

### Selection criteria

Studies were included in the analysis if they met the following inclusion criteria: (1) evaluation of the predictive effect of TMB in the outcome of ICIs in HNSCC patients; (2) TMB assessment before treatment; (3) TMB cutoff value was provided; (4) data availability of TMB-related hazard ratio (HR) and its corresponding 95% confidence interval (95% CI) for overall survival (OS), as well as the odds ratio (OR) and its corresponding 95% CI for the objective response rate (ORR) to ICIs; and (5) original articles published in English between January 2011 and December 2023.

The following exclusion criteria were also applied to the selected studies: (1) insufficient or lacking information related to TMB prognostic accuracy, including HRs or ORs with 95% CI and (2) article type classified as a letter, case report, non-clinical study, or conference abstract.

### Data extraction

The relevant information from each selected paper was independently extracted by two reviewers (JPR and MS-C), and any disagreements were resolved through consensus. The predetermined data from each article were documented as follows: Name of first author, year of publication, country, study population (number of patients with high or low TMB), study design, median age, median follow-up, treatment method, clinical stage, sample source (tumor or blood), sequencing method (NGS or WES), covariant, cut-off value of TMB, method of cut-off value determination, median TMB value, and survival analysis.

### Quality assessment

Two authors (JPR and MS-C) independently assessed the quality of the eligible studies by Newcastle–Ottawa Scale (NOS), using three parameters with a maximum of 9 points: comparability (0–4 points), selection (0–2 points), and outcome confirmation (0–3 points) [30]. A score greater than 6 points indicated a high-quality article, while a score of 6 points or lower indicated a low-quality article.

### Statistical analysis

Meta-analysis was performed using *Review Manager (RevMan) version 5.4* and package meta from the software environment R (www.r-project.ort). Significant results were defined as those having a *p*-value below 0.05. To assess the predictive efficacy of TMB in HNSCC patients treated with ICIs, the OS and ORR were compared between the high and low TMB patient subgroups using HRs and ORs. Forest plots were used to visualize the overall effect. A Dersimonian-Laird random-effect model was employed in order to deal with potential heterogeneity among the included studies. Statistical heterogeneity was assessed through visual examination of the forest plots, and its magnitude was quantified using the I-square and Chi-Square tests. Subgroup analysis was performed based on the sequencing method (NGS or WES), cut-off value (≥ 10 or < 10), and TMB quantification method (muts/Mb or muts/exome) in order to detect sources of heterogeneity and to analyze the results of populations with different characteristics. The leave-one-out procedure was used to complement the subgroup analysis for evaluating the sensitivity of the obtained results. Finally, publication bias was assessed by visual inspection of funnel plots and Egger’s test [31].

## Results

### Search results

Our search strategy led to a total of 2985 studies retrieved from PubMed, Web of Science, EMBASE and Scopus databases. However, 1960 of these studies were screened after removal of duplicates. We excluded animal studies, reviews, comments, case reports, studies unrelated to the topic and non-English language studies through title and abstract screening. This resulted in 49 studies that were further reviewed in detail. Following a thorough assessment by full-text review, we removed studies that were not clinical trials or cohort studies, as well as those lacking extractable data or utilizing different analytic parameters. Finally, 11 studies published between 2018 and 2023 were included in this meta-analysis (Fig. 1) [18–28].

### Study characteristics

See Additional file 1: Table S3 presents study characteristics, while Additional file 1: Table S4 provides a summary of the key findings. This meta-analysis included a total of 1200 patients, with each study ranging from 10 to 257 patients, and two studies [25, 27] including two different cohorts. Among the 11 included studies, seven studies were conducted in the USA, while single studies were performed in Japan, Italy and China, and another across multiple geographic areas.

This analysis consisted of four prospective studies and seven retrospective cohort studies. In terms of treatment approaches, all studies included ICI treatment (monotherapy or in combination). The survival data were reported as OS in four out of 11 studies, six studies presented data as ORR, and the remaining study reported both OS and ORR.

### Quality assessment

Based on NOS assessment, three studies achieved a score of 7, indicating intermediate-quality, while eight studies obtained scores ranging from 8 to 9, indicating high-quality (Additional file 1: Table S5).

### TMB and method of obtaining the cut-off value

TMB was determined through NGS/WES sequencing analyses of tumor samples in all the studies, except one study [20] that also assessed peripheral blood samples (Additional file 1: Table S3).

Different methods were used to establish the cut-off values for high and low TMB. The cut-off of muts/Mb values ranged from 2.54 to 20.0, with a mean of 8.14 and the muts/exome values ranged from 86.0 to 175.0, with a mean of 130.5. Data were reported in mutations per megabase (muts/Mb) in nine studies, and mutations per exome (mut/exome) in two studies [21, 26]. The median muts/Mb values ranged from 3.04 to 7.6, with a mean of 5.47. The muts/exome values were not available. One study defined a high TMB value of 175.0 muts/exome based on a literature review [21], and two studies used a cut-off of 10.0 and 15.0 muts/Mb [20, 28]. Three studies established cut-off values based on the median TMB, with calculated values of 5.0 [18], 6.71 [22], and 7.6 [23]. Another study determined the optimal value using the Cox proportional hazards model, setting 10 muts/Mb as a cut-off [25], while another one employed R language survival package analysis to determine a cut-off value of 2.54 [24]. In addition, one study determined the optimal cut-point using the Youden Index, setting it at 86.0 muts/exome [26], in another a threshold for TMB was chosen to attain the best performance in predicting progression free survival (PFS) in a univariable survival model, setting it at 3.34 muts/Mb [27], whereas in the remaining study the method used to obtain the cut-off value was not specified [19].

### Association between TMB and objective response rate after ICI treatment

**Fig. 2:**
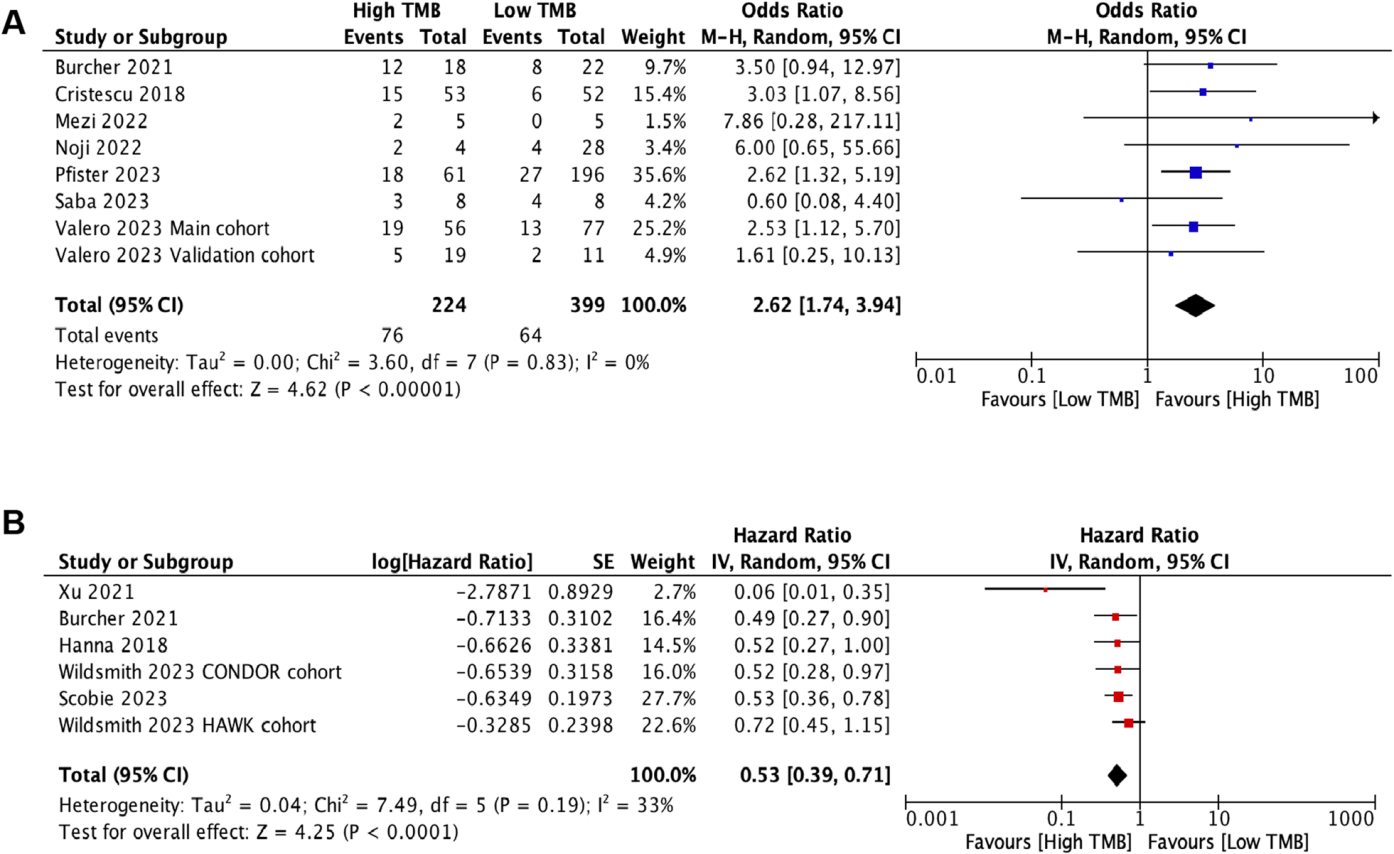
Forest plots of the meta-analysis in HNSCC patients treated with ICIs. Impact of TMB on ORR (**A**) and OS after ICI treatment (**B**)

The pooled ORR for ICI treatment was evaluated across seven studies (eight cohorts) involving 623 patients, using a random-effects model [18–22, 26, 27]. The group of patients with high TMB exhibited a superior ORR (OR = 2.62, 95% CI 1.74–3.94, *p* < 0.0001; Fig. 2A). Pooled data were homogenous under a random-effects model (*p* = 0.83, I^2^ = 0%).

### Association between TMB and overall survival in ICI-treated HNSCC patients

The impact of TMB on HNSCC survival after ICI treatment was assessed in five studies (six cohorts) involving 596 patients, using a random-effects model [18, 23–25, 28]. Results from these studies consistently showed a better survival in patients harboring high TMB. The group of patients with high TMB exhibited a survival advantage (HR = 0.53; 95% CI 0.39–0.71, *p* < 0.0001; Fig. 2B). Pooled data were homogenous under a random-effects model (*p* = 0.19, I^2^ = 33%).

### Heterogeneity and sensitivity analysis

Subgroup analysis was conducted to investigate the factors contributing to heterogeneity. No substantial alteration in heterogeneity was observed when subgroup analyses were performed based on sequencing method (NGS or WES), cut-off value (≥ 10 or < 10 muts/Mb), or TMB quantification (muts/Mb or muts/exome). These data are shown in Additional file 1: Table S6.

In addition, a sensitivity analysis was performed using the leave-one-out method to explore potential sources of heterogeneity among the 11 included studies and the robustness of the results. This analysis revealed that the exclusion of any single study did not lead to a significant alteration in the overall prevalence or heterogeneity. These results are included in Additional file 1: Figure S7.

### Published status bias analysis

**Fig. 3:**
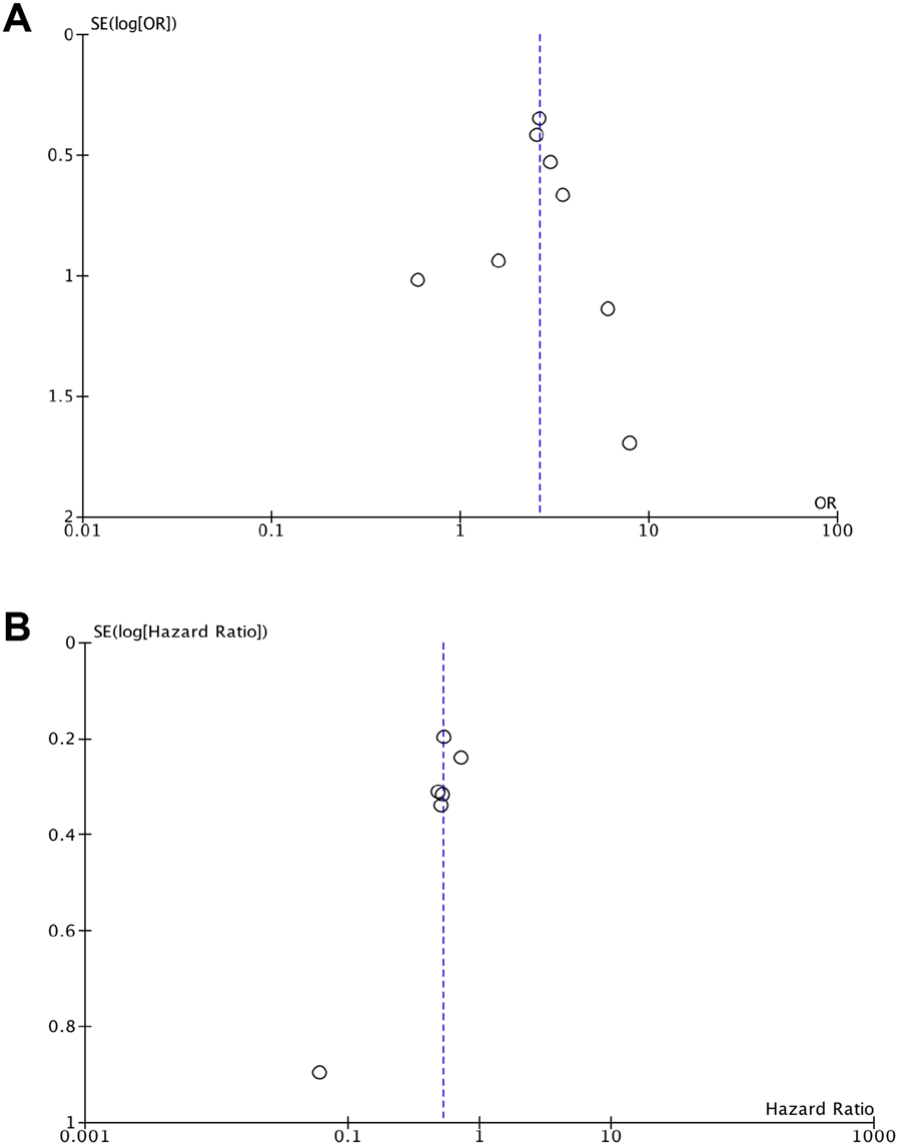
Funnel plots for the impact of TMB on ORR (**A**) and on OS after ICI treatment (**B**)

Publication bias in literature was assessed by funnel plot (Fig. 3) and Egger’s test. There was no evidence of publication bias for ORR after ICI-treated patients (Egger’s test: *p* = 0.9782). In the OS in ICI-treated patients we detect some publication bias (Egger´s test: *p* = 0.0461). This bias was mostly caused by Xu et al. [24], a small study with the largest effect. It is noteworthy that when this study was removed, the effect changed from 0.53 to 0.56 (Additional file 1: Figure S7). In addition, if we symmetrize the data (i.e. by including an artificial study with the same weight that Xu et al. [24] mirrored respect the average effect size), the obtained random-effect HR would be 0.55 [0.38–0.80] (Additional file 1: Figure S8).

## Discussion

This is the first meta-analysis specifically evaluating the prognostic and predictive significance of TMB in R/M HNSCC after ICI treatment. It includes a substantial number of patients (n = 1200) across 11 studies to consistently demonstrate that patients with a high TMB had a significant survival advantage, both in terms of OS and ORR. In addition, an elevated TMB was associated with a better response to ICI therapy. Thus, the risk of death was reduced by 47% in patients harboring high TMB, and the ORR was 2.62 times higher compared to the low TMB subgroup.

In the last few decades, TMB has been gradually gaining prominence as a prospective biomarker for immunotherapy response. Earlier research indicated a positive correlation between elevated TMB and increased tumor neoantigens displayed by major histocompatibility complex class (MHC) molecules. This, in turn, plays a role in promoting immune detection and enhancing the response to anti-tumor immunotherapies [32]. Numerous studies have demonstrated that TMB has the capacity to predict ICI efficacy across different tumor types [12–17, 33, 42] and also some meta-analyses that included few (< 3) HNSCC studies [34, 35]. A portion of TMB has the potential to induce the formation of neoantigens during tumor progression. As the tumor's TMB increases, the likelihood of neoantigen formation also rises, thus triggering the recognition and activation of T cells [32, 36]. This is exemplified by the direct correlation between the mean TMB and the objective response rate observed across 27 tumor types [37]. By contrast, the presence of numerous tumor subclones may lead to neoantigen heterogeneity and cause host immune invalidation despite harboring a high mutational load [38]. This could explain why some patients with high TMB do not respond to ICI therapy.

Based on our meta-analysis it seems that the detection of TMB could be helpful to improve the selection of HNSCC patients who may benefit from ICI therapy. However, there are still several challenges related to the clinical interpretation of TMB testing results, as highlighted by Ma et al. [39]. At present, there are no clear consensus methods for optimal cut-off determination of high TMB. This is evident in this meta-analysis since each of the included studies employed a different method to calculate the cut-off values for TMB. In addition, the number of mutations obtained in each study was variable and the cut-off of muts/Mb values ranged from 2.54 to 20.0, with a mean of 8.14, and the muts/exome values ranged from 86.0 to 175.0, with a mean of 130.5. The TMB values are heterogeneous among different cancer types, and an appropriate high TMB value has not been established for all solid tumors nor for each specific tumor subtype. Finally, the TMB testing method varied; most studies used NGS, but four studies used WES. Even though initial investigations into TMB relied on the WES approach, its practical use in clinical settings is limited by its high cost, long detection time, complex data interpretation, and the need for fresh samples. The rapid advancement of NGS technologies has enabled the acceleration of whole genome sequencing, due to its ultrahigh throughput, scalability, and speed. Several studies have also highlighted a significant correlation between TMB measured through WES and targeted panel sequencing [13, 40, 41]. However, differences in the targeted exome panels, sequencing depth, and bioinformatics algorithms could lead to heterogeneous results. Uniform industry standards for TMB testing by NGS are needed for clinical use [39].

These difficulties in defining the cut-off values and inconsistent detection platforms for TMB prompted the study by Oiu et al*.* [42] to search for an alternative biomarker. These authors found that comutation of the Spliceosome (Sp) pathway and Hedgehog (He) signaling pathway (defined as SpHe-comut +) was associated with increased TMB and neoantigen load, as well as increased levels of immune-related signatures. Furthermore, this study also revealed SpHe-comut + as an effective predictor of immunotherapeutic benefit, with an OR of 1.74 [1.74–2.15] for ORR and a HR of 0.76 [0.64–0.91] for OS after ICI treatment, which is similar to our reported results in ORR and OS. Therefore, SpHe-comut + has emerged as an optional and cost-effective approach to identify potential immunotherapy responders, and hence to improve patient stratification and treatment decision-making [42].

Another relevant issue that influences TMB detection is the origin of samples. Tissue-based TMB (tTMB) assessment is predominant in clinical settings. However, when tissue samples are insufficient, inadequate and/or clinically unfeasible for TMB measurement, blood-based TMB (bTMB) could be instead used to evaluate immunotherapy efficacy [13]. All but one study included in this meta-analysis used tissue-based TMB assessment, whereas the study performed by Noji et al*.* [20] included both tTMB and bTMB. Another important issue to consider for prediction of ICI effectiveness is the source of the tumor sample analyzed, due to the inconsistent status of various biomarkers in primary tumors and paired metastasis [43]. Notably, TMB and microsatellite instability status were less prone to change between primary tumors and their corresponding metastases. In marked contrast, PD-L1, PD-1, PD-L2, and tumor-infiltrating lymphocyte (TIL) density led to a higher frequency of discordance [43].

Our meta-analysis demonstrates the impact of TMB on the effectiveness of ICI treatment in R/M HNSCC patients. Nevertheless, there are still several limitations, such as the potential existence of publication bias suggested by our funnel plots, or unreported data, that cannot be ruled out. Moreover, some selected studies adopted a retrospective, non-randomized approach that could potentially amplify the impact of confounding factors. Furthermore, the potential application of TMB as a biomarker in clinical settings continues to be a subject of debate. Challenges persist in standardizing uniform TMB assessment procedures. Further prospective investigations are warranted to validate the optimal selection of TMB assessment platforms and targeted sequencing panels. The existing research on TMB as a predictive biomarker in HNSCC immunotherapy remains insufficient. Possible correlations between TMB and other predictive biomarkers require validation through large scale randomized trials, and establishment of the most effective predictive combination. Recent advancements in machine learning techniques, particularly artificial intelligence-based predictive analysis, offer a tremendous potential for the identification of TME-related biomarkers in HNSCC patients. This progress enables the analysis of extensive multi-omics datasets into transformative solutions to improve clinical decision-making and implement fundamental changes in the tumor treatment paradigm of patients with locally advanced or R/M HNSCC [44].

## Conclusion

The results of this unprecedented meta-analysis serve to demonstrate that HNSCC patients with high TMB exhibit a higher benefit from ICI-based therapy compared to the low TMB subgroup, hence suggesting that tumor mutation load could be a useful biomarker to predict ICI response and treatment efficacy in these patients. Together these findings should encourage further investigation of TMB assessment prior to immunotherapy in R/M HNSCC as a standardized and validated biomarker in prospective clinical trials.

### Supplementary Information

**Additional file 1: Table S1.** PRISMA 2020 Checklist. **Table S2.** Search strategy in PubMed**. Table S3.** Main features of the selected studies. **Table S4.** Key findings of the selected studies. **Table S5.** The Newcastle–Ottawa Scale (NOS) for assessing the quality of studies in meta-analyses. **Table S6.** Subgroup analyses of ORR and OS in HNSCC patients treated with ICIs. **Figure S7.** Leave-one-out sensitivity analysis of ORR and OS in HNSCC patients treated with ICIs. **Figure S8.** Forest plot of OS after symmetrizing the data in HNSCC patients treated with ICIs.

## Data Availability

The data collected during the systematic review are available on request.

## Abbreviations

bTMB: Blood-based TMB
HR: Hazard ratio
HNSCC: Head and neck squamous cell carcinoma
ICIs: Immune checkpoint inhibitors
MHC: Major histocompatibility complex class
Muts/exome: Mutations per exome
Muts/Mb: Mutations per megabase
NGS: Next-generation sequencing
NOS: Newcastle–Ottawa scale
ORR: Objective response rate
OR: Odds ratio
OS: Overall survival
PRISMA: Preferred reporting items for systematic reviews and meta-analyses
PFS: Progression free survival
R/M: Recurrent and metastatic
tTMB: Tissue-based TMB
TME: Tumor microenvironment
TMB: Tumor mutational burden
WES: Whole-exome sequencing
