# Supplementary material for: Tumor mutational burden predictability in head and neck squamous cell carcinoma patients treated with immunotherapy: systematic review and meta-analysis
Source: J Transl Med. 2024 Feb 4;22:135. doi: 10.1186/s12967-024-04937-x (PMC10840180; doi:10.1186/s12967-024-04937-x)

**SUPPLEMENTARY INFORMATION**

**Additional Table S1. PRISMA 2020 Checklist.**

| **Section and Topic** | **Item #** | **Checklist item** | **Location where item**  **is reported** |
| --- | --- | --- | --- |
| **TITLE** | | |  |
| Title | 1 | Identify the report as a systematic review. | Page 1 |
| **ABSTRACT** | | |  |
| Abstract | 2 | See the PRISMA 2020 for Abstracts checklist. | Page 2 |
| **INTRODUCTION** | | |  |
| Rationale | 3 | Describe the rationale for the review in the context of existing knowledge. | Page 3-4 |
| Objectives | 4 | Provide an explicit statement of the objective(s) or question(s) the review addresses. | Page 4 |
| **METHODS** | | |  |
| Eligibility criteria | 5 | Specify the inclusion and exclusion criteria for the review and how studies were grouped for the syntheses. | Page 5-6 |
| Information sources | 6 | Specify all databases, registers, websites, organisations, reference lists and other sources searched or consulted to identify studies. Specify the date when each source was last searched or consulted. | Page 5 |
| Search strategy | 7 | Present the full search strategies for all databases, registers and websites, including any filters and limits used. | Additional Table S2 |
| Selection process | 8 | Specify the methods used to decide whether a study met the inclusion criteria of the review, including how many reviewers screened each record and each report retrieved, whether they worked independently, and if applicable, details of automation tools used in the process. | Page 6 |
| Data collection process | 9 | Specify the methods used to collect data from reports, including how many reviewers collected data from each report, whether they worked independently, any processes for obtaining or confirming data from study investigators, and if applicable, details of automation tools used in the process. | Page 6 |
| Data items | 10a | List and define all outcomes for which data were sought. Specify whether all results that were compatible with each outcome domain in each study were sought (e.g. for all measures, time points, analyses), and if not, the methods used to decide which results to collect. | Page 5-6 |
|  | 10b | List and define all other variables for which data were sought (e.g. participant and intervention characteristics, funding sources). Describe any assumptions made about any missing or unclear information. | Page 6 |
| Study risk of bias assessment | 11 | Specify the methods used to assess risk of bias in the included studies, including details of the tool(s) used, how many reviewers assessed each study and whether they worked independently, and if applicable, details of automation tools used in the process. | Page 6 |
| Effect measures | 12 | Specify for each outcome the effect measure(s) (e.g. risk ratio, mean difference) used in the synthesis or presentation of results. | Page 6 |
| Synthesis methods | 13a | Describe the processes used to decide which studies were eligible for each synthesis (e.g. tabulating the study intervention characteristics and comparing against the planned groups for each synthesis (item #5)). | Page 6 |
|  | 13b | Describe any methods required to prepare the data for presentation or synthesis, such as handling of missing summary statistics, or data conversions. | - |
|  | 13c | Describe any methods used to tabulate or visually display results of individual studies and syntheses. | - |
|  | 13d | Describe any methods used to synthesize results and provide a rationale for the choice(s). If meta-analysis was performed, describe the model(s), method(s) to identify the presence and extent of statistical heterogeneity, and software package(s) used. | Page 7 |
|  | 13e | Describe any methods used to explore possible causes of heterogeneity among study results (e.g. subgroup analysis, meta-regression). | Page 7 |
|  | 13f | Describe any sensitivity analyses conducted to assess robustness of the synthesized results. | Page 7 |
| Reporting bias assessment | 14 | Describe any methods used to assess risk of bias due to missing results in a synthesis (arising from reporting biases). | Page 6 |
| Certainty assessment | 15 | Describe any methods used to assess certainty (or confidence) in the body of evidence for an outcome. | Page 7 |

| **Section and Topic** | **Item #** | **Checklist item** | **Location where item**  **is reported** |
| --- | --- | --- | --- |
| **RESULTS** | | |  |
| Study selection | 16a | Describe the results of the search and selection process, from the number of records identified in the search to the number of studies included in the review, ideally using a flow diagram. | Page 7-8 |
|  | 16b | Cite studies that might appear to meet the inclusion criteria, but which were excluded, and explain why they were excluded. | - |
| Study characteristics | 17 | Cite each included study and present its characteristics. | Page 8 |
| Risk of bias in studies | 18 | Present assessments of risk of bias for each included study. | Additional Table S5 |
| Results of individual studies | 19 | For all outcomes, present, for each study: (a) summary statistics for each group (where appropriate) and (b) an effect estimate and its precision (e.g. confidence/credible interval), ideally using structured tables or plots. | Figure 2A-2B |
| Results of syntheses | 20a | For each synthesis, briefly summarise the characteristics and risk of bias among contributing studies. | Additional Table S3-S4 |
|  | 20b | Present results of all statistical syntheses conducted. If meta-analysis was done, present for each the summary estimate and its precision (e.g. confidence/credible interval) and measures of statistical heterogeneity. If comparing groups, describe the direction of the effect. | Figure 2A-2B |
|  | 20c | Present results of all investigations of possible causes of heterogeneity among study results. | Page 10 |
|  | 20d | Present results of all sensitivity analyses conducted to assess the robustness of the synthesized results. | Page 10 |
| Reporting biases | 21 | Present assessments of risk of bias due to missing results (arising from reporting biases) for each synthesis assessed. | Figure 3 |
| Certainty of evidence | 22 | Present assessments of certainty (or confidence) in the body of evidence for each outcome assessed. | Page 9 |
| **DISCUSSION** | | |  |
| Discussion | 23a | Provide a general interpretation of the results in the context of other evidence. | Page 10 |
|  | 23b | Discuss any limitations of the evidence included in the review. | Page 11-12 |
|  | 23c | Discuss any limitations of the review processes used. | Page 12 |
|  | 23d | Discuss implications of the results for practice, policy, and future research. | Page 12-13 |
| **OTHER INFORMATION** | | |  |
| Registration and protocol | 24a | Provide registration information for the review, including register name and registration number, or state that the review was not registered. | Page 5 |
|  | 24b | Indicate where the review protocol can be accessed, or state that a protocol was not prepared. | Not prepared |
|  | 24c | Describe and explain any amendments to information provided at registration or in the protocol. | - |
| Support | 25 | Describe sources of financial or non-financial support for the review, and the role of the funders or sponsors in the review. | Page 15 |
| Competing interests | 26 | Declare any competing interests of review authors. | Page 16 |
| Availability of data, code and other materials | 27 | Report which of the following are publicly available and where they can be found: template data collection forms; data extracted from included studies; data used for all analyses; analytic code; any other materials used in the review. | Page 16 |

From *Page., et al* 2021 [29]

For more information, visit: <http://www.prisma-statement.org/>

**Additional Table S2. Search strategy.**

| **PUBMED:**  *tumor mutational burden AND (head and neck cancer OR oral cancer OR oropharyngeal cancer OR laryngeal cancer OR hypopharyngeal cancer) Sort by: Most Recent ("cysts"[MeSH Terms] OR "cysts"[All Fields] OR "cyst"[All Fields] OR "neurofibroma"[MeSH Terms] OR "neurofibroma"[All Fields] OR "neurofibromas"[All Fields] OR "tumor s"[All Fields] OR "tumoral"[All Fields] OR "tumorous"[All Fields] OR "tumour"[All Fields] OR "neoplasms"[MeSH Terms] OR "neoplasms"[All Fields] OR "tumor"[All Fields] OR "tumour s"[All Fields] OR "tumoural"[All Fields] OR "tumourous"[All Fields] OR "tumours"[All Fields] OR "tumors"[All Fields]) AND ("mutate"[All Fields] OR "mutated"[All Fields] OR "mutates"[All Fields] OR "mutating"[All Fields] OR "mutation"[MeSH Terms] OR "mutation"[All Fields] OR "mutations"[All Fields] OR "mutation s"[All Fields] OR "mutational"[All Fields] OR "mutator"[All Fields] OR "mutators"[All Fields]) AND ("burden"[All Fields] OR "burdened"[All Fields] OR "burdening"[All Fields] OR "burdens"[All Fields]) AND ("head and neck neoplasms"[MeSH Terms] OR ("head"[All Fields] AND "neck"[All Fields] AND "neoplasms"[All Fields]) OR "head and neck neoplasms"[All Fields] OR ("head"[All Fields] AND "neck"[All Fields] AND "cancer"[All Fields]) OR "head and neck cancer"[All Fields] OR ("mouth neoplasms"[MeSH Terms] OR ("mouth"[All Fields] AND "neoplasms"[All Fields]) OR "mouth neoplasms"[All Fields] OR ("oral"[All Fields] AND "cancer"[All Fields]) OR "oral cancer"[All Fields]) OR ("oropharyngeal neoplasms"[MeSH Terms] OR ("oropharyngeal"[All Fields] AND "neoplasms"[All Fields]) OR "oropharyngeal neoplasms"[All Fields] OR ("oropharyngeal"[All Fields] AND "cancer"[All Fields]) OR "oropharyngeal cancer"[All Fields]) OR ("laryngeal neoplasms"[MeSH Terms] OR ("laryngeal"[All Fields] AND "neoplasms"[All Fields]) OR "laryngeal neoplasms"[All Fields] OR ("laryngeal"[All Fields] AND "cancer"[All Fields]) OR "laryngeal cancer"[All Fields]) OR ("hypopharyngeal neoplasms"[MeSH Terms] OR ("hypopharyngeal"[All Fields] AND "neoplasms"[All Fields]) OR "hypopharyngeal neoplasms"[All Fields] OR ("hypopharyngeal"[All Fields] AND "cancer"[All Fields]) OR "hypopharyngeal cancer"[All Fields]))*  Date of search: 26 December 2023  Search results: 871  No limitations applied |
| --- |
| **WEB OF SCIENCE:**  *TS= (“tumor mutational burden” AND (“head and neck cancer” OR “oral cancer” OR “oropharyngeal cancer” OR “laryngeal cancer” OR “hypopharyngeal cancer”) (Topic) and 2023 or 2022 or 2021 or 2020 or 2019 or 2018 or 2017 or 2016 or 2015 or 2014 or 2012 or 2011 (Publication Years) and English (Languages))*  Date of search: 26 December 2023  Search results: 254  No limitations applied |
| **SCOPUS:**  *ALL (“tumor mutational burden” AND (“head and neck cancer” OR “oral cancer” OR “oropharyngeal cancer” OR “laryngeal cancer” OR “hypopharyngeal cancer”))*  Date of search: 26 December 2023  Search results: 504  No limitations applied |
| **EMBASE:**  *('tumor mutational burden'/exp OR 'tumor mutational burden' OR (('tumor'/exp OR tumor) AND mutational AND ('burden'/exp OR burden))) AND (('head'/exp OR head) AND ('cancer'/exp OR cancer) OR 'oral cancer'/exp OR 'oral cancer' OR (oral AND ('cancer'/exp OR cancer)) OR 'oropharyngeal cancer'/exp OR 'oropharyngeal cancer' OR (oropharyngeal AND ('cancer'/exp OR cancer)) OR 'laryngeal cancer'/exp OR 'laryngeal cancer' OR (laryngeal AND ('cancer'/exp OR cancer)) OR 'hypopharyngeal cancer'/exp OR 'hypopharyngeal cancer' OR (hypopharyngeal AND ('cancer'/exp OR cancer)))*  Date of search: 26 December 2023  Search results: 1356  All fields searched  No limitations applied |

| **References** | **Year of publication** | **Country** | **Number of Patients (High/Low TMB)** | **Design of study** | **Median age (years)** | **Median follow-up (months)** | **Treatment** | **Clinical Stage** | **Sample Source** | **Sequencing Method** | **Covariant** | **Cut-off value of TMB** | **Method of obtaining the cut-off value** | **Median TMB (Range)** | **Statistic analysis** | **Score of NOS** |
| --- | --- | --- | --- | --- | --- | --- | --- | --- | --- | --- | --- | --- | --- | --- | --- | --- |
| Burcher et al., 2021[18] | 2021 | USA | 128 (58/70) | Retrospective | 60 | 20.5 | ICIs PD1/PD-L1 | R/M | Tumoral | NGS | High TMB | 5 Muts/Mb | Median | 5.0 (NA) | ORR/OS | 8 |
| Mezi et al., 2022 [19] | 2022 | Italy | 10 (2/8) | Prospective | 66 | 27 | Nivolumab | R/M | Tumoral | NGS | High TMB | 20 Muts/Mb | NA | NA | ORR | 9 |
| Noji et al., 2022 [20] | 2022 | Japan | 32 (4/29) | Retrospective | 60 | NA | Pembrolizumab/Nivolumab | R/M | Tumoral/blood (1 patient) | NGS | Top Tertile | 10 Muts/Mb | Review of literature | NA | ORR | 7 |
| Pfister et al.,2023 [21] | 2023 | USA | 257 (61/196) | Prospective (Phase Ib and II) | NA | NA | Pembrolizumab | R/M | Tumoral | WES | High TMB | 175 Muts/exome | Review of literature | NA | ORR | 7 |
| Saba et al., 2023 [22] | 2023 | USA | 16 (8/8) | Prospective (Phase II) | 62 | 10.6 | Pembrolizumab/Cabozantinib | R/M | Tumoral | WES | High TMB | 6.71 Muts/Mb | Median | 6.71 (0.39-10.66) | ORR | 9 |
| Hanna et al., 2018 [23] | 2018 | USA | 81 (NA) | Retrospective | 57 | 8.6 | ICIs PD-L1 | R/M | Tumoral | NGS | High TMB | 7.6 Muts/Mb | Median | 7.6 (1.5-76) | OS | 9 |
| Xu et al., 2021 [24] | 2021 | China | 39 (28/11) | Retrospective | 57 | NA | ICIs PD-1 | R/M | Tumoral | NGS | High TMB | 2.54 Muts/Mb | R language survival package analysis | NA | OS | 7 |
| Wildsmith et al., 2023 [25] | 2023 | UK, USA, Poland, Italy, France, Spain | 78 [HAWK cohort] | Retrospective | 60 | 6.1 | Durvalumab | R/M | Tumoral | WES | High TMB | 10 Muts/Mb | Cox proportional hazards model | NA | OS | 9 |
| Wildsmith et al., 2023 [25] | 2023 | UK, USA, Poland, Italy, France, Spain | 48 [CONDOR cohort] | Retrospective | 61 | 6.5 | Durvalumab+tremelimumab | R/M | Tumoral | WES | High TMB | 10 Muts/Mb | Cox proportional hazards model | NA | OS | 9 |
| Cristescu R et al., 2018 [26] | 2018 | USA | 126 (68/58) | Prospective (Phase Ib) | NA | NA | Pembrolizumab | R/M | Tumoral | WES | High TMB | 86 Muts/exome | Youden Index cut points | NA | ORR | 8 |
| Valero C et al., 2023 [27] | 2023 | USA | 133 (NA) [Main cohort] | Retrospective | 62 | 24 | ICIs PD1/PD-L1/CTLA-4 | R/M | Tumoral | WES | High TMB | 3.34 Muts/Mb | Univariable survival model | 3.04 (NA) | ORR | 9 |
| Valero C et al., 2023 [27] | 2023 | USA | 30 (NA) [Validation cohort] | Retrospective | NA | 24 | ICIs PD1/PD-L1 | R/M | Tumoral | NGS | High TMB | NA | NA | NA | ORR | 9 |
| Scobie et al., 2023 [28] | 2023 | USA | 222 (46/176) | Retrospective | 66.8 | 22.4 /24.9 | ICIs PD1/PD-L1 and combination | R/M | Tumoral | NGS | High TMB | 10 Muts/Mb | Review of literature | 5.0 (3.0-9.0) | OS | 9 |

**Additional Table S3. Main features of the selected studies**

**Abbreviations**

**TMB** Tumor Mutational Burden, H-TMB (High **NOS** Newcastle–Ottawa Scale, **ICIs** Immune checkpoint inhibitors, **R/M** Recurrent and metastatic, **WES** Whole-exome sequencing, **NGS** Next-generation sequencing

**OS** Overall survival, **ORR** Objective response rate

**NA** Not available

| **References** | **Key findings** |
| --- | --- |
| Burcher et al., 2021[18] | Patients with high TMB fared better in univariate and multivariate survival analysis. No correlation was found between PD-L1 expression and prognosis. There was a statistically significant association between PFS and response to IO and TMB. |
| Mezi et al., 2022 [19] | Our data confirm that an increased TMB may be associated with a greater sensitivity to immunotherapeutic agents. |
| Noji et al., 2022 [20] | The relationship between treatment response and oncogene profiling data in ICI-treated HNSCC patients was examined, and long-term response was observed in TMB-high cases. |
| Pfister et al.,2023 [21] | TMB and the inflammatory biomarkers PD-L1 and TcellinfGEP, assessed alone or together, may be useful for characterizing clinical response to pembrolizumab in R/M HNSCC. |
| Saba et al., 2023 [22] | There was no correlation between TMB level and response to therapy. |
| Hanna et al., 2018 [23] | Higher TMB among virus-negative SCCHN tumors predict anti–PD-1/L1 response. |
| Xu et al., 2021 [24] | High mutation burden and immune cell infiltration can improve the prognosis of HNSCC patients with immunotherapy, while of HNSCC patients with immunotherapy, while there was no significant effect on the efficacy. |
| Wildsmith et al., 2023 [25] | Findings support TMB as a biomarker for predicting survival in patients with platinum-resistant R/M HNSCC treated with ICIs. |
| Cristescu R et al., 2018 [26] | Tumor mutational burden (TMB) and a T cell-inflamed gene expression profile (GEP) exhibited joint predictive utility in identifying responders and nonresponders to the PD-1 antibody pembrolizumab. |
| Valero C et al., 2023 [27] | These findings shed light on the immunogenomic characteristics of HNSCC tumors that drive differential response to ICB and identify a clinical-genomic classifier that outperforms the current clinically approved biomarker of TMB. |
| Scobie et al., 2023 [28] | Consistent with previous studies, a predictive value of TMB ≥10 mut/Mb for ICI response was found in NSCLC and H&N, but not in esophageal/gastric cancer. |

**Additional Table S4. Key findings of the selected studies**

**Additional Table S5. The Newcastle-Ottawa Scale (NOS) for assessing the quality of studies in meta-analyses**

|  | **Selection** | | | | **Comparability** | **Exposure** | | | **Total Quality Score** |
| --- | --- | --- | --- | --- | --- | --- | --- | --- | --- |
| **References** | Representativeness of the exposed cohort | Selection of the non-exposed cohort | Ascertainment of exposure | Demonstration that outcome of interest was not present at start of study | Comparability of cohorts on the basis of the design or analysis | Assessment of outcome | Was follow-up long enough for outcomes to occur | Adequacy of follow up of cohorts |  |
| Burcher et al., 2021[18] | 1 | 1 | 1 | 1 | 1 | 1 | 1 | 1 | 8 |
| Mezi et al., 2022 [19] | 1 | 1 | 1 | 1 | 2 | 1 | 1 | 1 | 9 |
| Noji et al., 2022 [20] | 1 | 1 | 1 | 1 | 2 | 1 | 0 | 0 | 7 |
| Pfister et al.,2023 [21] | 1 | 1 | 1 | 1 | 2 | 1 | 0 | 0 | 7 |
| Saba et al., 2023 [22] | 1 | 1 | 1 | 1 | 2 | 1 | 1 | 1 | 9 |
| Hanna et al., 2018 [23] | 1 | 1 | 1 | 1 | 2 | 1 | 1 | 1 | 9 |
| Xu et al., 2021 [24] | 1 | 1 | 1 | 1 | 2 | 1 | 0 | 0 | 7 |
| Wildsmith et al., 2023 [25] | 1 | 1 | 1 | 1 | 2 | 1 | 1 | 1 | 9 |
| Cristescu R et al., 2018 [26] | 1 | 1 | 1 | 1 | 2 | 1 | 0 | 1 | 8 |
| Valero C et al., 2023 [27] | 1 | 1 | 1 | 1 | 2 | 1 | 1 | 1 | 9 |
| Scobie et al., 2023 [28] | 1 | 1 | 1 | 1 | 2 | 1 | 1 | 1 | 9 |

**Additional Table S6. Subgroup analyses of ORR and OS in HNSCC patients treated with ICIs.**

| **Subgroup analyses of ORR** |  | **No. of studies** | **Effects model** | **OR (95% CI)** | **P** | **Heterogeneity** | |
| --- | --- | --- | --- | --- | --- | --- | --- |
|  |  |  |  |  |  | **I^2^ (%)** | **P** |
| Sequencing method | NGS | 4 | Random | 3.36 (1.33-8.47) | 0.01 | 0 | 0.77 |
|  | WES | 4 | Random | 2.47 (1.56-3.89) | 0.0001 | 0 | 0.55 |
| Test for overall effect and for subgroup differences | | | | 2.62 (1.74-3.89) | <0.00001 | 0 | 0.56 |
| Muts/Mb Cut-off value | ≥ 10 | 2 | Random | 6.52 (1.03-41.47) | 0.05 | 0 | 0.89 |
|  | <10 | 3 | Random | 2.32 (1.14-4.70) | 0.02 | 9 | 0.33 |
| Test for overall effect and for subgroup differences | | | | 2.63 (1.42-4.87) | 0.002 | 4.8 | 0.31 |
| Ways to quantify mutations | Muts/Mb | 6 | Random | 2.50 (1.40-4.49) | 0.002 | 0 | 0.62 |
|  | Muts/exome | 2 | Random | 2.74 (1.55-4.85) | 0.0006 | 0 | 0.82 |
| Test for overall effect and for subgroup differences | | | | 2.62 (1.74-3.94) | <0.00001 | 0 | 0.83 |
|  | | | |  |  |  |  |
| **Subgroup analyses of OS** |  | **No. of studies** | **Effects model** | **HR (95% CI)** | **P** | **Heterogeneity** | |
|  |  |  |  |  |  | **I^2^ (%)** | **P** |
| Sequencing method | NGS | 4 | Random | 0.46 (0.29-0.71) | 0.005 | 45 | 0.14 |
|  | WES | 2 | Random | 0.64 (0.44-0.93) | 0.02 | 0 | 0.41 |
| Test for overall effect and for subgroup differences | | | | 0.53 (0.39-0.71) | <0.0001 | 22 | 0.26 |
| Muts/Mb Cut-off value | ≥ 10 | 3 | Random | 0.64 (0.44-0.93) | 0.02 | 0 | 0.41 |
|  | <10 | 3 | Random | 0.37 (0.17-0.81) | 0.01 | 61 | 0.08 |
| Test for overall effect and for subgroup differences | | | | 0.51 (0.34-0.77) | 0.001 | 33.7 | 0.22 |

**Additional Figure S7. Leave-one-out sensitivity analysis of ORR (a) and OS (b) in HNSCC patients treated with ICIs.**

**a)**

**b)**
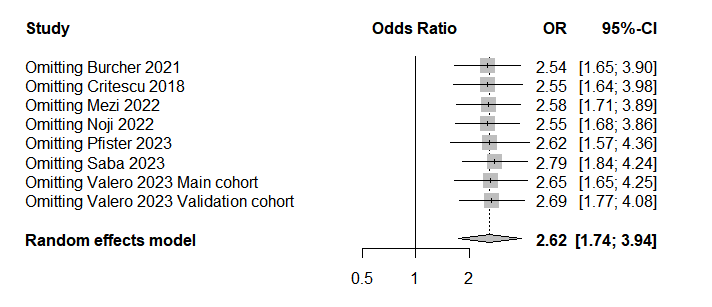


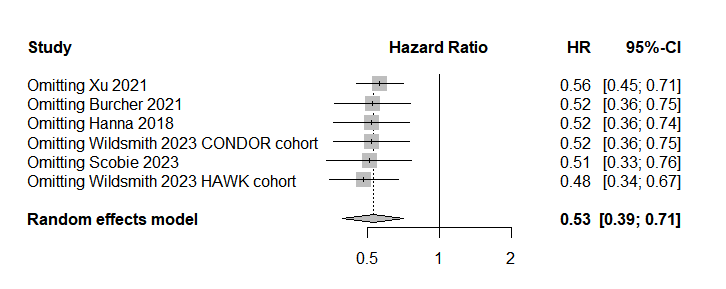


**Additional Figure S8. Forest plot of OS after symmetrizing the data in HNSCC patients treated with ICIs.**


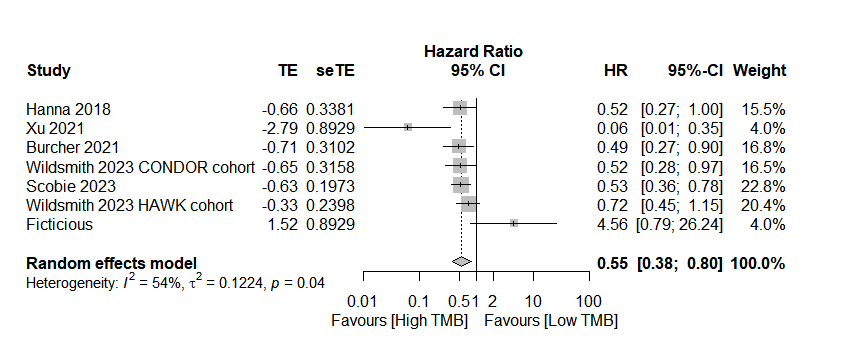

Supplement: Supplementary file 1 — Additional file 1: Table S1. PRISMA 2020 Checklist. Table S2. Search strategy in PubMed. Table S3. Main features of the selected studies. Table S4. Key findings of the selected studies. Table S5. The Newcastle–Ottawa Scale (NOS) for assessing the quality of studies in meta-analyses. Table S6. Subgroup analyses of ORR and OS in HNSCC patients treated with ICIs. Figure S7. Leave-one-out sensitivity analysis of ORR and OS in HNSCC patients treated with ICIs. Figure S8. Forest plot of OS after symmetrizing the data in HNSCC patients treated with ICIs. [file 12967_2024_4937_MOESM1_ESM.docx]
